# Genetic Diagnosis and Family Cascade Screening for Familial Hypercholesterolaemia in Patients with Premature Coronary Artery Disease: A Prospective Cohort Study from Vietnam

**DOI:** 10.3390/jpm16080403

**Published:** 2026-07-28

**Authors:** Sy Van Hoang, Kha Minh Nguyen, Hai Phuong Nguyen Tran, Hung Phi Truong, Tai Nhat Nguyen, Sang Quang Ly

**Affiliations:** 1 Department of Internal Medicine, School of Medicine, University of Medicine and Pharmacy at Ho Chi Minh City, 217 Hong Bang Street, Cho Lon Ward, Ho Chi Minh City 700000, Vietnam; hoangvansy@ump.edu.vn (S.V.H.); truongphihung2007@ump.edu.vn (H.P.T.); nguyennhattai@ump.edu.vn (T.N.N.); lyquangsang@ump.edu.vn (S.Q.L.); 2 Department of Cardiology, Cho Ray Hospital, Ho Chi Minh City 700000, Vietnam; 3 Department of Interventional Cardiology, Cho Ray Hospital, Ho Chi Minh City 700000, Vietnam; tnphuonghaibvcr@gmail.com

**Keywords:** familial hypercholesterolaemia, personalized medicine, cascade screening, genetic testing, premature coronary artery disease, LDL receptor, precision medicine

## Abstract

**Background**: Familial hypercholesterolaemia (FH) is a common monogenic cause of premature coronary artery disease (CAD), yet data from South-East Asia are sparse and the yield of family cascade screening has not previously been reported in Vietnam. **Methods**: In this prospective, single-centre cohort study (March 2023–September 2025), 500 consecutive patients with premature CAD (men < 55 years, women < 60 years) underwent targeted next-generation sequencing of LDLR, APOB and PCSK9, with Sanger confirmation of pathogenic or likely pathogenic (P/LP) variants. First-degree relatives of variant-positive index patients were offered structured counselling and cascade sequencing of the family-specific variant. **Results**: A P/LP variant was identified in 18 of 500 patients (3.6%; 95% CI 2.1–5.4%); 17 (94.4%) involved LDLR and one APOB. Nine distinct P/LP variants were observed, with several recurrent, consistent with possible founder effects. Two carriers were homozygous, including one with an APOB frameshift attributable to consanguinity. Variant carriers were younger (mean difference −5.6 years, 95% CI −9.2 to −2.0) with higher LDL-cholesterol (median 227.5 vs. 138 mg/dL) and more extensive coronary disease than non-carriers. Cascade screening was completed in 20 of 26 eligible families (77%); among 105 first-degree relatives tested, 46 (43.8%; 95% CI 34.7–53.4%) carried the family-specific variant, most of them young, asymptomatic offspring or siblings. **Conclusions**: Precise molecular diagnosis in a small number of index patients identified a much larger cohort of at-risk relatives who would otherwise have remained undiagnosed, providing the first Vietnamese evidence that hospital-anchored cascade screening is feasible and high-yielding in a resource-limited setting.

## 1. Introduction

Familial hypercholesterolaemia (FH) is among the most common monogenic disorders in humans, with an estimated global prevalence of one in 313 [1]. It is most frequently caused by pathogenic variants in the low-density lipoprotein receptor gene (LDLR), and less often in apolipoprotein B (APOB) or proprotein convertase subtilisin/kexin type 9 (PCSK9) [2,3]. These variants impair receptor-mediated clearance of low-density lipoprotein cholesterol (LDL-C) and produce a lifelong elevation of plasma LDL-C, generating an atherogenic burden that is far greater than the burden of comparable, acquired dyslipdaemia. Untreated heterozygous FH is associated with an approximately three- to four-fold increase in the risk of coronary artery disease (CAD), and the prevalence of the disorder is substantially enriched among patients presenting with early-onset events, reaching 6.7% among individuals with premature ischaemic heart disease in a global meta-analysis [1,4].

FH is a paradigmatic setting for a personalized-medicine approach to cardiovascular disease. Every genetic diagnosis is simultaneously (i) an individually informative event for the index patient, the identification of a pathogenic variant sharpens diagnostic certainty, refines cardiovascular risk stratification, and provides a mechanistic rationale for early, intensive and lifelong LDL-C lowering [3,5]; and (ii) a family-level event, because each first-degree relative has an a priori 50% probability of carrying the same variant, and can be tested rapidly by a single-variant, low-cost assay [6]. Cascade screening, testing that radiates from a genetically confirmed index case outward through relatives, is therefore endorsed by international guidelines as the most clinically effective and cost-effective strategy for detecting FH, and its use is increasingly viewed as a benchmark implementation of precision medicine in cardiovascular care [2,6,7,8].

Despite the effectiveness of statins and, more recently, of PCSK9 inhibitors, FH remains profoundly underdiagnosed worldwide, with fewer than 10% of affected individuals identified [5,9]. The gap is even wider in low- and middle-income countries (LMICs), including much of South-East Asia. Diagnostic genetic testing is not routinely available; costs are typically borne by patients; specialist genetic counsellors are scarce; national FH registries do not exist; reimbursement pathways for testing and for advanced lipid-lowering therapy are largely absent; and contemporary data on the molecular spectrum of FH and on the yield and feasibility of cascade screening in these populations are limited [10,11,12]. This evidence gap constrains policy: without local data, health systems cannot rationally allocate resources for genetic testing, cannot judge which patients to prioritize, and cannot design an FH pathway that fits the operational realities of local cardiology practice.

In Vietnam, a pioneering pediatric FH registry has begun to characterize homozygous and heterozygous FH in children [12], and a small case series from our group has reported the prevalence of genetically diagnosed FH in patients with premature acute myocardial infarction [13]. However, the diagnostic yield and feasibility of family cascade screening in the adult premature-CAD population have not previously been reported. Addressing this gap is essential if a practicable, personalized-medicine FH pathway is to be designed for Vietnam and comparable settings.

We therefore conducted a prospective, single-centre study at a Vietnamese tertiary cardiovascular centre with three aims: first, to determine the prevalence and molecular spectrum of LDLR/APOB/PCSK9 variants in a large cohort of patients with premature CAD; second, to characterize the clinical phenotype of variant carriers compared with non-carriers; and third, to quantify the diagnostic yield and feasibility of a hospital-anchored family cascade screening programme. The model tested, anchoring cascade screening in an existing cardiology service that already cares for patients with premature CAD, was chosen deliberately for its operational fit with the realities of cardiovascular care in resource-limited settings and its potential to convert individual genetic diagnoses into personalized family-level intervention.

## 2. Materials and Methods

### 2.1. Study Design and Population

This was a prospective, single-centre study conducted at Cho Ray Hospital, a national tertiary cardiovascular referral centre in Ho Chi Minh City, Vietnam, between March 2023 and September 2025. The study comprised two linked components: cross-sectional genetic characterization of consecutive patients with premature CAD (the index cohort) and prospective family cascade screening of their first-degree relatives.

Consecutive patients aged 18 years or older were eligible if they had premature CAD, defined as age at presentation under 55 years in men and under 60 years in women, together with either an acute coronary syndrome or a chronic coronary syndrome and angiographically confirmed obstructive disease (luminal stenosis of at least 50% in one or more of the three major epicardial coronary arteries on invasive or computed tomography coronary angiography). Patients who did not consent to participation and genetic counselling were excluded.

### 2.2. Clinical Assessment

Demographic data, cardiovascular risk factors, family history, anthropometric measurements, physical signs of lipid accumulation (tendon xanthoma and arcus cornealis), a fasting lipid profile and the angiographic extent of CAD (including the Gensini score) were recorded using a standardized case-report form. Clinical features relevant to FH were documented for every patient. LDL-C was measured directly at the Chợ Rẫy Hospital biochemistry laboratory before initiation or intensification of any lipid-lowering therapy where feasible; when patients were already receiving statin therapy at first assessment, the recorded LDL-C reflects the on-treatment value.

### 2.3. Genetic Testing and Variant Classification

Genomic DNA was obtained from peripheral blood or buccal mucosal swabs. Targeted next-generation sequencing (NGS) was performed using the GenLDL panel provided by the Medical Genetics Institute (Gene Solutions, Ho Chi Minh City, Vietnam), which captures the coding regions and exon–intron boundaries of *LDLR*, *APOB*, and *PCSK9*. Paired-end sequencing was carried out on an Illumina NextSeq 550 platform. Reads were aligned to the GRCh38 reference genome. The mean depth of coverage for the targeted regions was approximately 300× (minimum coverage threshold 30×), consistent with the manufacturer’s standard analytical performance for the panel. Copy-number variant (CNV) analysis of *LDLR* was performed by NGS read-depth analysis to detect exon-level deletions and duplications. Deep intronic, promoter and untranslated regions were not systematically captured by the panel.

Detected variants were interpreted according to the standards of the American College of Medical Genetics and Genomics (ACMG), incorporating mode of inheritance, population allele frequency, in silico prediction and the ClinVar database, and were assigned to one of five categories: pathogenic, likely pathogenic, variant of uncertain significance (VUS), likely benign or benign [14,15]. All pathogenic or likely pathogenic (P/LP) variants identified by NGS were subsequently confirmed by Sanger sequencing in the index patient before cascade testing. Patients carrying a P/LP variant were considered to have genetically confirmed FH. To contribute reference data from an under-represented population, variants identified in this cohort were submitted to the public ClinVar database.

### 2.4. Family Cascade Screening Protocol

Once a P/LP variant was confirmed in an index patient, that patient and their first-degree relatives were invited for structured pre-test genetic counselling. Counselling was provided by cardiologists formally trained in FH-focused genetic counselling, using a standardized information sheet and a study-specific written consent form. Because all screened families resided outside metropolitan Ho Chi Minh City, cascade samples were collected exclusively during outreach visits to families’ home provinces (i.e., 100% of screened families required outreach; on-site sampling at Cho Ray Hospital was not required). First-degree relatives (parents, siblings, and offspring) underwent buccal-swab sampling and targeted single-variant Sanger sequencing for the specific variant identified in the corresponding index patient. Relatives found to carry the family variant were referred back to their local cardiology services and, where appropriate, to Cho Ray Hospital, for lipid assessment and management in accordance with current guidelines [7].

Twenty-six index patients were eligible for cascade screening; 20 families (76.9%) completed cascade testing and 6 (23.1%) declined participation, most frequently citing concerns about the cost of testing, the time required for outreach visits and perceived insurance implications of genetic diagnosis.

### 2.5. Outcomes

The primary outcome was the diagnostic yield of cascade screening, defined as the proportion of tested first-degree relatives carrying the same FH-related variant as their index case. Secondary outcomes were the prevalence and molecular spectrum of FH-related variants in the index cohort; clinical, laboratory and angiographic differences between variant carriers and non-carriers; the distribution of newly identified carriers by kinship; and the uptake of first-line lipid-lowering therapy among all identified P/LP-variant carriers.

### 2.6. Statistical Analysis

Continuous variables are presented as mean ± standard deviation or median with interquartile range, according to distribution, and categorical variables as counts and percentages. Carriers and non-carriers were compared using the independent-samples *t*-test or the Mann–Whitney U test for continuous variables, and the chi-squared or Fisher exact test for categorical variables. For all key comparisons, effect sizes with 95% confidence intervals (mean differences for continuous variables; odds ratios for categorical variables) are reported in addition to *p*-values. Wilson 95% confidence intervals were computed for proportions. Comparisons between carriers and non-carriers, as well as the LDL-C cutoff analysis, are exploratory; *p*-values were not adjusted for multiplicity and should be interpreted accordingly. To evaluate the practical utility of LDL-C as a triage tool for genetic testing, we computed the sensitivity, specificity and positive predictive value (PPV) of a series of LDL-C thresholds (≥130, ≥155, ≥190, ≥250 and ≥300 mg/dL) for identifying P/LP-variant carriers in the index cohort. Data were managed in Microsoft Excel 2021 (Microsoft Corp., Redmond, WA, USA) and analyzed with Stata/MP version 17.0 (StataCorp LLC, College Station, TX, USA).

### 2.7. Ethical Approval

The study was conducted in accordance with the Declaration of Helsinki and approved by the Biomedical Research Ethics Committee of Cho Ray Hospital (Decision No. 1508/GCN-HDDD, approved 9 March 2023). All participants provided written informed consent before enrolment, genetic testing and cascade screening.

## 3. Results

### 3.1. Index Cohort

Five hundred consecutive patients with premature CAD were enrolled. The mean age was 46.8 ± 6.1 years, and 410 patients (82.0%) were men. All patients underwent next-generation sequencing of LDLR, APOB and PCSK9.

### 3.2. Prevalence and Molecular Spectrum of FH-Related Variants

An FH-related gene variant was detected in 26 of 500 patients (5.2%). A P/LP variant, defining genetically confirmed FH, was identified in 18 patients (3.6%; 95% CI, 2.1–5.4%). Of these, 17 (94.4%) involved LDLR, and one (5.6%) involved APOB; no pathogenic PCSK9 variant was detected. A further eight patients (1.6%) carried a variant of uncertain significance. LDL-C in P/LP-variant carriers ranged from 94 to 441 mg/dL (median 227.5, IQR 192–304), and in non-carriers from 14 to 318 mg/dL (median 138, IQR 108–166).

Nine distinct P/LP variants were identified (Table 1). Several were recurrent: the LDLR missense variant c.664T>C (p.Cys222Arg) was carried by five unrelated index patients and c.311G>A (p.Cys104Tyr) by three; three further variants each occurred in two patients. This clustering of a small number of variants is consistent with possible founder effects in the local population.

### 3.3. Two Homozygous Cases

Case 1—LDLR c.664T>C (p.Cys222Arg) homozygous. A 39-year-old woman presented with a long-standing history of severe cardiovascular disease. She had undergone coronary artery bypass grafting for three-vessel coronary disease at age 26 and mechanical aortic valve replacement (Bentall procedure) for severe aortic stenosis at age 37. Physical examination showed multiple tendinous xanthomas over the metacarpophalangeal and interphalangeal joints of both hands (typical of homozygous FH). Her baseline lipid profile, on ongoing high-intensity rosuvastatin (20 mg daily), was total cholesterol 496 mg/dL, LDL-C 440.7 mg/dL, HDL-cholesterol 50 mg/dL and triglycerides 73 mg/dL. There was no reported parental consanguinity. Despite this classical clinical picture, a formal diagnostic label of familial hypercholesterolaemia and referral to a lipid clinic had not been made prior to the current study, and no cascade family screening had ever been offered. This case illustrates that homozygous or compound-heterozygous FH may remain unrecognized even after decades of severe atherosclerotic cardiovascular disease and multiple cardiac surgeries when a formal molecular diagnosis is not sought.

Case 2—APOB c.44_50del (p.Pro15fs) homozygous. A 41-year-old woman presented with premature acute coronary syndrome. She was the offspring of a first-cousin marriage, and autozygosity for this rare frameshift variant is the most plausible explanation for homozygosity. There was no history of premature coronary disease in the immediate family known to the patient, and no prior lipid clinic contact. Baseline LDL-C at presentation was 195 mg/dL; arcus cornealis was present, but tendon xanthomata were absent. She has since been treated with atorvastatin 40 mg daily and ezetimibe 10 mg daily.

### 3.4. Clinical and Laboratory Characteristics of Variant Carriers

The 18 P/LP-variant carriers presented on average almost six years earlier than non-carriers, had substantially higher LDL-C and more extensive coronary disease (Table 2). Effect sizes with 95% confidence intervals are reported to convey the magnitude and precision of associations in addition to *p*-values, and should be interpreted as exploratory given the small carrier subgroup and the number of comparisons performed.

### 3.5. LDL-Cholesterol Thresholds for Triggering Genetic Testing

Because Reviewer feedback prompted us to evaluate LDL-C-based triage more formally, we computed the operating characteristics of five LDL-C thresholds for identifying P/LP-variant carriers in the index cohort (Table 3). Even at a low threshold of ≥130 mg/dL, sensitivity for detecting P/LP-variant carriers was only 90%; no threshold captured all carriers, because the lowest observed LDL-C among carriers was 94 mg/dL. The conventional adult FH threshold of ≥190 mg/dL detected 70% of carriers with a specificity of 88% (PPV 12.5%); lowering to ≥155 mg/dL raised sensitivity to 80% at the cost of specificity (68%) and PPV (5.8%).

These observations have two practical implications. First, an LDL-C threshold of ≥190 mg/dL, as recommended by ESC/EAS guidance, is a specific but insensitive trigger for genetic testing in a premature CAD population and would miss a clinically important fraction of P/LP-variant carriers. Second, no single LDL-C threshold identifies all carriers; six of eighteen carriers in our cohort had LDL-C values below the classical ≥190 mg/dL threshold, and one carrier had LDL-C below 100 mg/dL. This underscores the added value of gene-based diagnosis over an LDL-C-only strategy, particularly in patients whose LDL-C may already be attenuated by prior statin therapy or by other modifying factors, and it supports offering genetic testing to premature-CAD patients across a broader LDL-C spectrum than that captured by the conventional adult FH cutoff.

### 3.6. Outcomes of Family Cascade Screening

**Figure 1:**
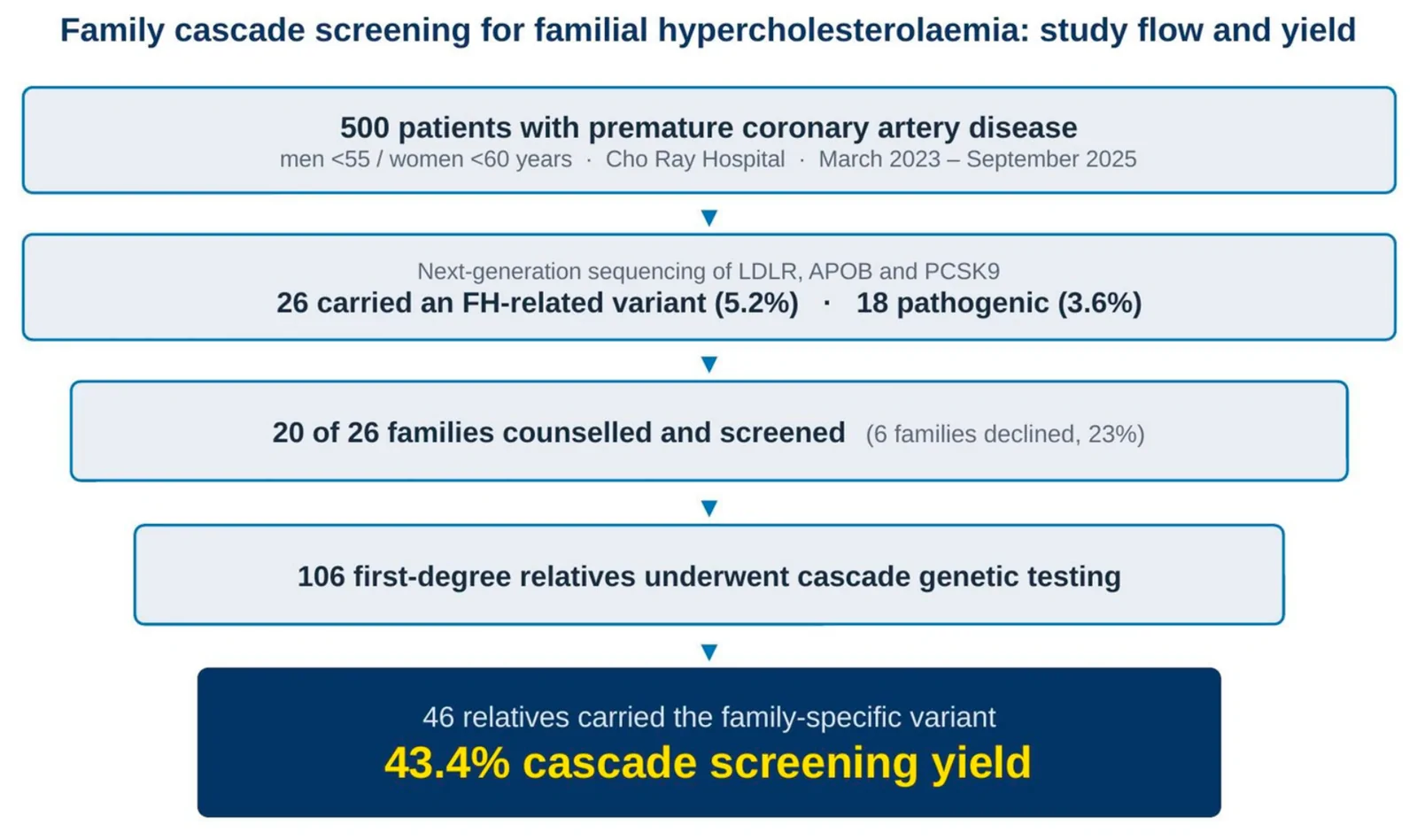
Study flow and yield of family cascade screening. Of 500 patients with premature coronary artery disease, 26 carried an FH-related gene variant; 20 of their families completed cascade screening (six declined participation) and 105 first-degree relatives were tested, of whom 46 (43.8%) carried the family-specific variant.

Of 26 index families eligible for cascade screening (i.e., families of the 26 index patients carrying any FH-related variant, including VUS), 20 (76.9%) completed cascade testing and 6 (23.1%) declined participation. Across the completed pedigrees, 105 first-degree relatives (parents, siblings and offspring) underwent buccal-swab sampling and targeted single-variant Sanger sequencing (median five relatives per family, range 1 to 22). The primary outcome, the proportion of tested first-degree relatives carrying the family-specific variant, was 43.8% (46 of 105; 95% CI 34.7–53.4%), closely matching the 50% expected for an autosomal dominant trait (Figure 1, Table 4). Newly identified carriers were predominantly the offspring and siblings of the index patient; the majority were young and had no known cardiovascular disease at the time of testing. Because families were dispersed across multiple provinces, all cascade sample collection was performed during outreach visits (100% of 20 screened families), highlighting both the reach and the logistical demands of a hospital-anchored cascade model in a resource-limited setting. Variants identified in this cohort were deposited in the public ClinVar database to contribute reference data from an underrepresented Asian population [16,17].

### 3.7. Post-Diagnosis Management

All 18 P/LP-variant index patients were established on high-intensity statin therapy (100%; atorvastatin 40–80 mg or rosuvastatin 20–40 mg daily), and all had been initiated on adjunctive ezetimibe 10 mg daily. None of the identified carriers—index cases or relatives—had access to PCSK9 inhibitor therapy: at the time of the study, PCSK9 inhibitors were not reimbursed by the Vietnamese national health insurance system, and no FH-specific reimbursement pathway existed for advanced lipid-lowering therapy. Detailed lipid-lowering outcomes in newly identified relatives were beyond the scope of this observational study and are the subject of ongoing follow-up.

## 4. Discussion

In this prospective study of 500 patients with premature CAD in Vietnam, genetically confirmed FH was present in 3.6% of index patients, and family cascade screening identified the family-specific variant in 43.8% of first-degree relatives tested. To our knowledge, this is the first report of the yield and feasibility of family cascade screening for FH among adults in Vietnam, and it operationalizes a personalized-medicine approach to a common inherited cardiovascular disorder in a resource-limited South-East Asian setting.

### 4.1. Prevalence, Molecular Spectrum and Generalizability

The 3.6% prevalence of P/LP variants in our index cohort is consistent with international series of patients with early-onset coronary disease, in which genetically confirmed FH has been reported in approximately 2–9% [1,4], and is more than ten times the background population estimate of one in 313 [1]. Because our index cohort was recruited at a single national tertiary referral centre and restricted to patients with angiographically confirmed obstructive coronary disease, the cohort is enriched for more severe disease phenotypes. This case-mix bias probably inflates the observed prevalence relative to what would be observed among all patients presenting with symptoms suggestive of CAD in primary or secondary care, and the yield of a comparable cascade programme in community-based cohorts may therefore be lower. The findings should be interpreted as informative for tertiary cardiology services managing patients with confirmed premature CAD, and should be replicated in broader Vietnamese and regional cohorts before national extrapolation.

An earlier and smaller report from our group examined the prevalence of genetically diagnosed FH in a partly overlapping cohort of patients with premature acute myocardial infarction [13]; that report addressed prevalence only, its index cohort is partly subsumed within the present larger series, and it did not include family cascade screening. The present study extends those observations to a substantially larger and broader cohort and adds the cascade-screening dimension that has not previously been reported in Vietnam.

The variant spectrum was dominated by LDLR (94.4%), in keeping with reports from other populations [3,11]. The recurrence of a small number of variants—most notably LDLR c.664T>C (p.Cys222Arg) in five unrelated families and c.311G>A (p.Cys104Tyr) in three—raises the possibility of founder effects in the local population. If confirmed in larger series, such founder variants could enable a simplified, low-cost, population-specific genotyping panel as a first-line diagnostic and screening tool. This is a strategy that would be particularly well-suited to LMIC settings, where it could substantially reduce the cost, complexity, and turnaround time of FH diagnosis.

### 4.2. Homozygous FH Remains Missed Even After Decades of Severe Disease

The two homozygous cases described here illustrate two important messages. First, homozygous FH may remain formally unrecognized for decades, even in patients with unambiguous clinical stigmata and repeated encounters with cardiology services. The 39-year-old woman with homozygous LDLR c.664T>C had already undergone coronary artery bypass grafting at age 26 and Bentall aortic root replacement at age 37, and displayed multiple tendinous xanthomas, yet had not been formally diagnosed with FH or offered cascade screening until enrolment in this study. This is consistent with international data indicating that fewer than 10% of individuals with FH are diagnosed [5,9], and reinforces the importance of embedding molecular FH testing into routine premature-CAD pathways.

Second, molecular findings in consanguineous families require careful interpretation. The 41-year-old woman with homozygous APOB c.44_50del was the offspring of a first-cousin marriage; autozygosity for this rare frameshift variant is the most plausible mechanism for homozygosity. Her LDL-C of 195 mg/dL is lower than the LDL-C values typically observed in classical homozygous FH, but is nonetheless above the standard adult threshold for FH and, in the context of premature acute coronary syndrome, autozygosity for a rare APOB variant and consanguinity support a clinically relevant contribution of the variant. Consanguinity remains prevalent in several regions of Vietnam and across South-East Asia, and clinicians should retain a high index of suspicion for rare recessive-appearing variants of familial dyslipidaemia in this setting.

### 4.3. Genotype–Phenotype Associations and the Case for a Broader LDL-C Trigger

Patients with genetically confirmed FH presented, on average, almost six years earlier than non-carriers and had markedly higher LDL-C, more frequent stigmata of lipid accumulation and a heavier, more diffuse burden of coronary atherosclerosis. These observations are biologically coherent: a pathogenic variant confers lifelong elevation of LDL-C and a correspondingly greater cumulative exposure to atherogenic lipoprotein than acquired dyslipidaemia [4]. They also have a direct clinical message. Identification of a pathogenic variant supports early initiation of high-intensity statin therapy, combination lipid-lowering treatment with ezetimibe and, where accessible, PCSK9 inhibition, together with aggressive LDL-C goals and lifelong follow-up [7,11]. Genetic diagnosis has been shown to be an independent predictor of cardiovascular events beyond clinical scoring in large FH cohorts [18].

Our formal cutoff analysis (Table 3) has an important clinical implication. Although the conventional adult FH threshold of ≥190 mg/dL is highly specific, it is insensitive in a premature-CAD population and misses approximately one in three P/LP-variant carriers. Lowering the threshold to ≥155 mg/dL captures a greater fraction of carriers at the cost of specificity, and no LDL-C threshold captured all carriers in our cohort. This finding supports offering genetic testing to premature-CAD patients across a broader LDL-C range than that captured by the conventional cutoff, particularly where LDL-C values may already be attenuated by ongoing statin therapy at presentation, and is directly relevant to the design of pragmatic FH testing pathways in cardiology services in resource-limited settings.

### 4.4. Family Cascade Screening: Turning Individual Diagnosis into Family Level Intervention

The central finding of this study is the high yield of cascade screening. A 43.8% positivity rate among first-degree relatives closely matches the theoretical 50% for an autosomal dominant disorder and is comparable with cascade programmes reported from established high-income settings [6,7,19]. In practical terms, precise genetic diagnosis in a small number of index patients identified an additional cohort of high-risk first-degree relatives, most of them young and asymptomatic, who would otherwise have remained undiagnosed. This is a textbook illustration of personalized medicine: individual-level genotypic information, coupled with a structured, pedigree-based testing strategy, transforms one clinical encounter into an intervention that protects an entire kindred [6,7].

Cascade screening is consistently identified as the most clinically effective and cost-effective approach to FH case-finding and is strongly endorsed by international guidelines [2,7,12,20]. In the SAFEHEART registry, molecularly defined FH conferred a substantially elevated cardiovascular risk that was reduced when patients were identified and treated early [18]; the majority of newly identified carriers in our study, young offspring and siblings of the index patient, represent precisely the population in whom early lipid-lowering therapy can prevent premature events and preserve a near-normal cardiovascular trajectory [9].

Our post-diagnosis management data provide a candid picture of what can, and cannot, currently be delivered in Vietnam. All 18 index carriers were on high-intensity statin therapy plus ezetimibe. No carrier had access to PCSK9 inhibitor therapy; however, these agents are not reimbursed by the Vietnamese national health insurance system, and no FH-specific reimbursement pathway exists. Bridging the gap between molecular diagnosis and access to full-spectrum lipid-lowering therapy will therefore be the next central challenge for personalized FH care in Vietnam and in similar LMIC settings, and is a policy question that goes beyond the reach of any single clinical study.

### 4.5. Implications for FH Care in Low- and Middle-Income Countries

**Figure 2:**
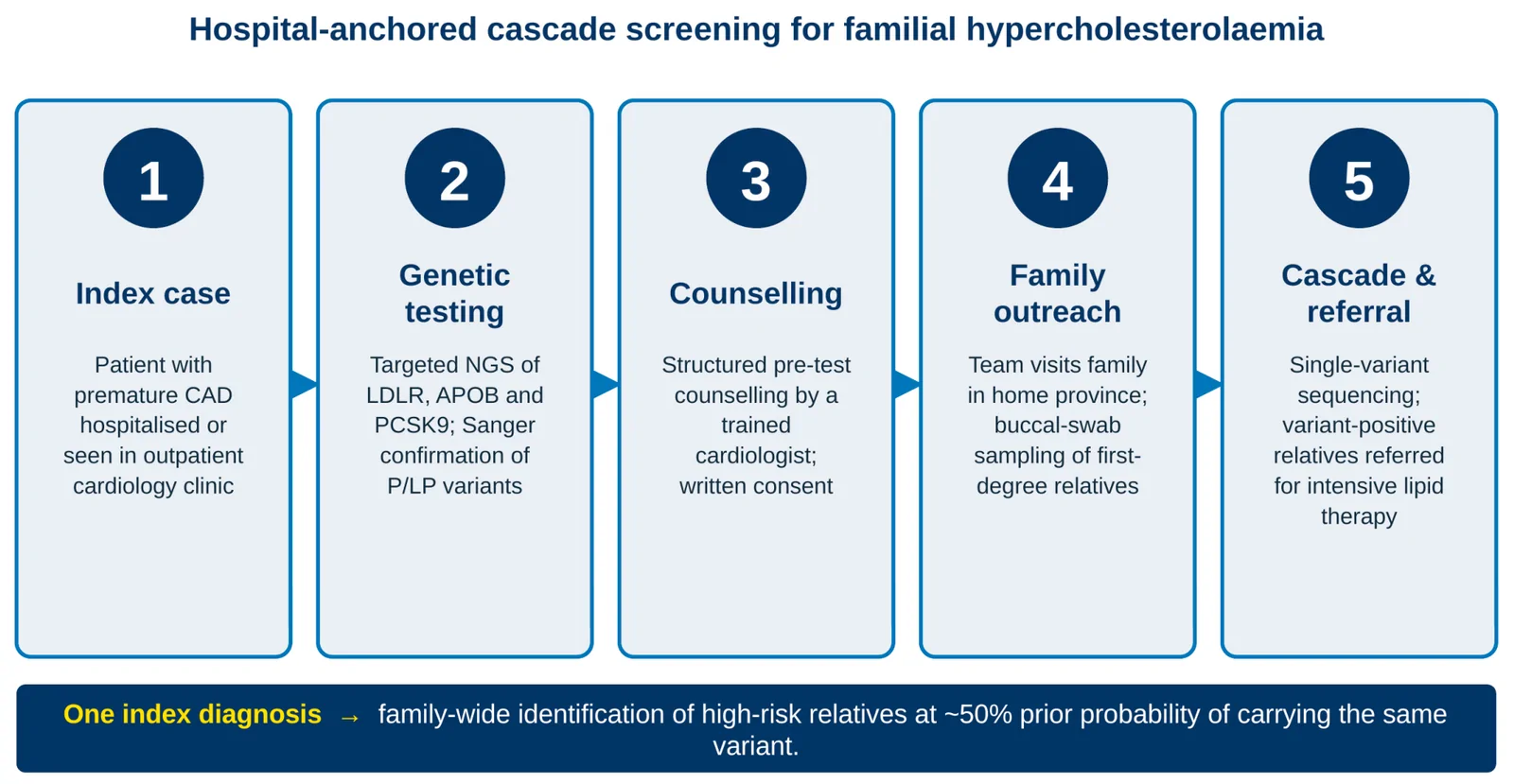
Hospital-anchored cascade screening workflow for familial hypercholesterolaemia in a resource-limited setting: identification of the index case during premature-CAD hospitalization, on-site genetic counselling, outreach visits to home provinces for buccal-swab sampling, targeted single-variant sequencing and referral of variant-positive relatives for lipid management.

Several LMIC-specific implications deserve emphasis. First, population-wide genetic screening for FH is not financially or operationally realistic in most LMIC health systems. Diagnostic genetic testing is not part of standard cardiology pathways; costs are typically borne by patients; dedicated genetic counsellors are scarce; and national registries and reimbursement mechanisms are largely absent [10,11,12]. A hospital-anchored cascade model, in which the index case is identified at the time of a hospitalization for premature CAD (when motivation for diagnosis and treatment is at its peak), and in which testing radiates only to relatives at a 50% a priori probability of carrying the same variant, offers a pragmatic, high-yield alternative [6]. The model uses the existing cardiology workforce, leverages a captive index population enriched for FH, and targets diagnostic resources at the relatives most likely to benefit. Figure 2 illustrates this workflow in a form intended to be transferable to other cardiology services in the region.

Second, our data speak to global genetic equity. Variant databases underpinning clinical interpretation remain heavily biassed toward populations of European ancestry, and Asian and South-East Asian variants are substantially underrepresented, generating a higher proportion of ambiguous or uncertain interpretations in patients from these populations [16,17]. Depositing variants identified in this cohort in ClinVar contributes reference data from a population that is currently poorly represented and, together with the observation of several recurrent LDLR variants, provides a basis for population-specific reference data and the eventual design of a targeted Vietnamese genotyping panel.

Third, participation was incomplete: six of 26 eligible families (23%) declined cascade screening, and all screened families required outreach sampling in their home provinces. This mirrors the international observation that the principal barriers to cascade screening are not biological but social and structural: concerns about insurance and employment discrimination, the direct and indirect costs of testing, the burden of travel from rural to urban centres, limited public awareness of FH and a paucity of trained counsellors [7,19]. Several of these barriers are amenable to system-level intervention. Community outreach for sample collection, the use of buccal-swab kits to reduce travel, structured counselling embedded within cardiology services and, over the longer term, public reimbursement of genetic testing and intensive lipid-lowering therapy for genetically confirmed FH are measures that have improved participation in other settings and that could realistically be piloted in Vietnam.

### 4.6. A Stepwise Agenda for FH Care in Vietnam

Taken together, these findings support a stepwise national agenda. In the short term, cardiology services that already manage patients with premature CAD can implement opportunistic FH testing for clear high-risk phenotypes (very high LDL-C, premature family history, physical stigmata), followed by cascade screening of the families of confirmed cases, the model that we have shown to be feasible. In the medium term, the spectrum of variants observed here should be expanded through multi-centre collaboration, and a low-cost targeted genotyping panel based on the recurrent variants identified could be evaluated as a first-line screening tool. In the longer term, formal recognition of FH as a routine entity in cardiovascular care will require national clinical guidance, reimbursement of genetic testing and advanced lipid-lowering therapy, public awareness campaigns, and structured training of cardiologists and genetic counsellors.

### 4.7. Strengths and Limitations

The strengths of this study include prospective, consecutive enrolment of a large index cohort, uniform next-generation sequencing of the three principal FH genes, systematic ACMG-based variant classification, Sanger confirmation of P/LP variants, a real-world cascade screening programme conducted in a middle-income setting with active community outreach, and the reporting of effect sizes with 95% confidence intervals in addition to *p*-values.

Several limitations should be acknowledged. First, the study was conducted at a single tertiary referral centre, and the index population was restricted to patients with angiographically confirmed obstructive CAD, which limits generalizability to community and primary-care settings. Second, genetic profiling of the index cohort was cross-sectional and long-term cardiovascular outcomes were not assessed. Third, cascade screening was limited to first-degree relatives; extension to more distant relatives would be expected to identify further carriers. Fourth, six of 26 eligible families declined participation, and thecharacteristics of non-participating families could not be compared systematically, so participation bias cannot be excluded. Fifth, deep intronic, promoter and untranslated regions were not covered by the panel, so a proportion of FH may remain undetected. Sixth, segregation and functional studies of variants of uncertain significance were not performed. Seventh, comparisons between carriers and non-carriers were exploratory, based on a small carrier subgroup and were not adjusted for multiplicity. Finally, the clinical management and lipid-lowering outcomes of newly identified relatives are the subject of ongoing follow-up and are not reported here. The broadest context possible. Future research directions may also be highlighted.

## 5. Conclusions

In a Vietnamese cohort of patients with premature CAD, genetically confirmed FH was common, and family cascade screening identified the disease-causing variant in 43.8% of first-degree relatives, the majority of them young, asymptomatic individuals. Precise molecular diagnosis in a small number of index patients thus identified a much larger group of high-risk relatives who would otherwise have remained undiagnosed. These findings provide the first Vietnamese evidence that a hospital-anchored, cascade screening model is feasible and high-yielding in a resource-limited setting, and support the integration of genetic testing and cascade screening into routine cardiovascular care as an operational model of personalized medicine for a common inherited cardiovascular disorder in Vietnam and similar low- and middle-income countries.

## Figures and Tables

**Table 1:** Pathogenic and likely pathogenic variants identified in the index cohort.

Gene	cDNA Change	Protein Change	Variant Type	ACMG Classification	Index Patients, *n*
*LDLR*	c.81C>A	p.Cys27Ter	Nonsense	Pathogenic	1
*LDLR*	c.311G>A	p.Cys104Tyr	Missense	Pathogenic	3
*LDLR*	c.664T>C	p.Cys222Arg	Missense	Pathogenic	5
*LDLR*	c.682G>A	p.Glu228Lys	Missense	Pathogenic	2
*LDLR*	c.1721G>A	p.Arg574His	Missense	Pathogenic	2
*LDLR*	c.1864G>C	p.Asp622His	Missense	Pathogenic	1
*LDLR*	c.2044C>T	p.Leu682Phe	Missense	Pathogenic	1
*LDLR*	c.2050G>A	p.Ala684Thr	Missense	Likely pathogenic	2
*APOB*	c.44_50del	p.Pro15fs	Frameshift	Pathogenic	1

ACMG, American College of Medical Genetics and Genomics. Two patients carried homozygous variants (LDLR c.664T>C and APOB c.44_50del) and the remainder were heterozygous. All P/LP variants identified by NGS were confirmed by Sanger sequencing before cascade testing.

**Table 2:** Clinical and laboratory characteristics of patients with premature coronary artery disease, by carriage of a pathogenic or likely pathogenic FH variant.

Characteristic	Variant Carriers (*n* = 18)	Non-Carriers (*n* = 482)	*p*-Value
Age, years	41.4 ± 7.6	47.0 ± 6.0	<0.001
Male sex	15 (83.3)	395 (82.0)	0.88
Current smoking	10 (55.6)	303 (62.9)	0.82
Hypertension	6 (33.3)	257 (53.3)	0.09
Diabetes mellitus	3 (16.7)	99 (20.5)	0.68
Family history of premature CAD	6 (33.3)	54 (11.2)	0.005
Body mass index, kg/m^2^	25.3 ± 2.7	24.2 ± 3.0	0.12
Physical signs of lipid accumulation	6 (33.3)	36 (7.5)	<0.001
LDL-cholesterol, mg/dL	227.5 (192–304)	138 (108–166)	<0.001
Triglycerides, mg/dL	172 (131–298)	191 (131–304)	0.68
Acute coronary syndrome presentation	13 (72.2)	385 (79.9)	0.43
Left main coronary disease	5 (27.8)	17 (3.5)	<0.001
Three-vessel disease	13 (72.2)	130 (26.9)	<0.001
Gensini score	90.5 (37.5–119.0)	50.8 (35.5–73.5)	<0.001

Values are mean ± standard deviation, median (interquartile range) or *n* (%). CAD, coronary artery disease; LDL, low-density lipoprotein.

**Table 3:** Operating characteristics of LDL-cholesterol thresholds for identifying pathogenic/likely pathogenic FH-variant carriers among 500 patients with premature CAD.

LDL-C Threshold, mg/dL	Sensitivity	Specificity	PPV
≥130	90%	51%	4%
≥155	80%	68%	6%
≥190	70%	88%	13%
≥250	30%	98%	30%
≥300	20%	99%	40%

CAD, coronary artery disease; FH, familial hypercholesterolaemia; LDL-C, low-density lipoprotein cholesterol; PPV, positive predictive value. Analysis restricted to patients with available baseline LDL-C.

**Table 4:** Family cascade screening outcomes.

Outcome	Value
Index families eligible for cascade screening	26
Families completing cascade screening, *n* (%)	20 (77)
Families declining participation, *n* (%)	6 (23)
First-degree relatives tested, *n*	105
Relatives tested per screened family, median (range)	5 (1–22)
Relatives carrying the family-specific variant, *n* (%)	46 (43.8)
95% confidence interval for the carrier yield	34.7–53.4%
Sample collection performed by outreach visits, *n* (%)	20 (100)

## Data Availability

The data supporting the findings of this study are available from the corresponding author upon reasonable request.

## Abbreviations

The following abbreviations are used in this manuscript:

ACMG	American College of Medical Genetics and Genomics
APOB	Apolipoprotein B
CAD	Coronary artery disease
CI	Confidence interval
CNV	Copy-number variant
FH	Familial Hypercholesterolaemia
LDL-C	Low-density lipoprotein cholesterol
LDLR	Low-density lipoprotein receptor
LMIC	Low- and middle-income country
MD	Mean difference
NGS	Next-generation sequencing
L/LP	Pathogenic or likely pathogenic
PPV	Positive predictive value
PCSK9	Proprotein convertase subtilisin/kexin type 9
VUS	Variant of uncertain significance.
